# The impact of FAP imaging in lung cancer and beyond: a new chapter

**DOI:** 10.1007/s00330-023-10398-5

**Published:** 2023-11-09

**Authors:** Emil Novruzov, Yuriko Mori, Abass Alavi, Frederik L. Giesel

**Affiliations:** 1 Department of Nuclear Medicine, Medical Faculty and University Hospital Duesseldorf, Heinrich-Heine-University Duesseldorf, 40225 Düsseldorf, Germany Moorenstrasse 5,; 2 Division of Nuclear Medicine, Department of Radiology, Hospital of the University of Pennsylvania, 3400 Spruce St., Philadelphia, PA 19104 USA

Despite the widespread screening measures and imaging methods with their steadily increasing diagnostic accuracy, lung cancer remains one of the most lethal malignancies worldwide. Given the limitations of conventional structural imaging modalities, the introduction of FDG-PET/CT made a great leap forward due to enhanced glycolytic activity of cancer cells known as the Warburg effect [1]. Combined FDG-PET/CT has enhanced the role of imaging in staging and re-staging lung cancer, guiding the type of therapy and therapy response monitoring [1, 2]. However, FDG-PET imaging is suboptimal due to its low specificity in making a distinction between inflammatory and malignant disorders, and requires hours of fasting. Furthermore, diabetes and other chronic diseases and disorders lead to substantially reduced accuracy due to reduced glucose uptake at the disease sites [1, 2]. In view of such limitations of FDG imaging, attempts have been made to introduce novel radiotracers based on fibroblast activation protein inhibitors (FAPI), which appear to be highly promising and effective in assessing cancer as well as inflammatory-fibrosing processes. This concept relies on the distinctive role of the tumor stroma in the cancer cell growth and progress by intense interaction between tumor cells and various elements of extracellular matrix (ECM), particularly fibroblasts [2, 3].

Although the exact mechanism is not yet fully understood, certain conditions such as acute and chronic inflammation and fibrosis are known to trigger fibroblast activation [3–5]. The activated fibroblasts are termed as cancer-associated fibroblasts (CAF) and arise from healthy resident tissue fibroblasts, bone marrow, and mesenchymal, epithelial, and endothelial cells. These are the main pro-tumorigenic factor in tumor stroma of epithelial malignancies, which modulate the tumor growth as well as inflammatory-fibrosing processes by expressing several surface biomarkers like TGF-β or FAP-α, among which fibroblast activation protein (FAP) being the most relevant target for diagnostic and theranostic purposes. Besides, this close similarity of fibroblast activity in tumorous and inflammatory processes explains the background for referring to malignancy as a “not-healing wound” [5, 6].

FAP, a type II transmembrane glycoprotein on CAFs, significantly influences ECM through enzymatic functions. Overexpressed in more than 90% of epithelial cancers and inflammatory-fibrosing processes, FAP is upregulated in lung cancer as well, varying by subtype. While NSCLC shows FAP expression levels of up to 100%, small cell lung cancer (SCLC) and large cell neuroendocrine carcinoma (LCNC) are known to express FAP biomarker in up to 67% [7]. Aggressive tumors correlate with increased FAP expression, indicating poor outcomes. FAP’s absence in normal tissues underscores its value for theranostic and diagnostic purposes [2, 6]. Novel FAP ligands act via inhibiting the enzymatic activity of FAP (endopeptidase activity), leading to ^68^ Ga- and ^18^F-labeled FAPI radiotracers for PET imaging. FAPI PET/CT, requiring no special preparation, offers a promising pan-cancer tool, suitable for therapeutic use with isotopes like ^177^Lu, ^225^Ac, or ^90^Y [8].

In the light of highly promising theranostic potential of FAPI imaging, Qiao et al conducted a single-center, retrospective study to deepen the insight in the field of FAPI imaging for the differential diagnosis of inflammatory and malignant pulmonary lesions [9]. The number of equivocal lesions and histopathological subtypes of malignant nodules as well as inflammatory lesions seems to be evenly distributed over the patient cohort, providing a reliable comparison supported by rigorous statistical assessment. Firstly, the authors assess tracer uptake of FDG and FAPI in the lesions by comparing semiquantitative parameters and conduct further analysis of diagnostic accuracy with the ROC curve analysis. Consistent with prior research, the authors found the semiquantitative parameters of SUV_max_ and SUV_mean_ of both tracers to be comparable. Notably, FAPI demonstrated a significantly greater lesion-to-background ratio, providing excellent background contrast in comparison to FDG [9].

The research group of Qiao et al described, to the first time, statistically different levels of FAPI uptake in distinct inflammatory pulmonary lesions. Namely, the statistical analysis displayed significant differences upon tracer uptake of lobar pneumonia, post-obstructive pneumonia, and bronchiectasis, the latter showing the most intense uptake. Nevertheless, inflammatory pulmonary lesions demonstrated lower tracer uptake than malignant lesions [10]. As the authors underline, dual-tracer imaging would be a viable option, especially in the setting of malignant pulmonary diseases with confounding inflammatory processes and, beyond that, certain pitfalls may be encountered through acquiring delayed images or semiquantitative differential analysis of trace uptake in the region of interest with FAPI imaging [6, 10].

In summary, with the introduction of FAP ligands, the imaging community has the promise of a novel and, probably, disruptive imaging probe that is a strong candidate to compensate for the limitations of FDG imaging in both oncological and, beyond that, fibrosing imaging of the lung owing to tumor stroma–specific imaging even with an option of theranostic application. Hence, FAP imaging has been evolving to the highlight of hybrid imaging in both research and clinical care of pulmonary diseases in the near future.

## Declarations

### Guarantor

Prof. Dr. Frederik L. Giesel.

### Statistics and biometry

No complex statistical methods were necessary for this paper.

### Study subjects or cohorts overlap

N/A

### Methodology

• Commentary
